# Investigating the effect of autograft diameter for quadriceps and patellar tendons use in anterior cruciate ligament reconstruction: a biomechanical analysis using a simulated Lachman test

**DOI:** 10.3389/fsurg.2023.1122379

**Published:** 2023-10-11

**Authors:** Farid Amirouche, Giovanni Francesco Solitro, Brandon Zachary Gligor, Mark Hutchinson, Jason Koh

**Affiliations:** ^1^ Department of Orthopaedics, University of Illinois at Chicago College of Medicine, Chicago, IL, United States; ^2^ Department of Orthopaedics, Northshore University Health System, Evanston, IL, United States; ^3^ Department of Orthopaedics, Louisiana State University College of Medicine, Shreveport, LA, United States

**Keywords:** ACL, autograft, patellar tendon, quadriceps tendon, diameter, finite element modeling, Lachman test

## Abstract

**Introduction:**

Current clinical practice suggests using patellar and quadriceps tendon autografts with a 10 mm diameter for ACL reconstruction. This can be problematic for patients with smaller body frames. Our study objective was to determine the minimum diameter required for these grafts. We hypothesize that given the strength and stiffness of these respective tissues, they can withstand a significant decrease in diameter before demonstrating mechanical strength unviable for recreating the knee's stability.

**Methods:**

We created a finite element model of the human knee with boundary conditions characteristic of the Lachman test, a passive accessory movement test of the knee performed to identify the integrity of the anterior cruciate ligament (ACL). The Mechanical properties of the model's grafts were directly obtained from cadaveric testing and the literature. Our model estimated the forces required to displace the tibia from the femur with varying graft diameters.

**Results:**

The 7 mm diameter patellar and quadriceps tendon grafts could withstand 55–60 N of force before induced tibial displacement. However, grafts of 5.34- and 3.76-mm diameters could only withstand upwards of 47 N and 40 N, respectively. Additionally, at a graft diameter of 3.76 mm, the patellar tendon experienced 234% greater stiffness than the quadriceps tendon, with similar excesses of stiffness demonstrated for the 5.34- and 7-mm diameter grafts.

**Conclusions:**

The patellar tendon provided a stronger graft for knee reconstruction at all diameter sizes. Additionally, it experienced higher maximum stress, meaning it dissociates force better across the graft than the quadriceps tendon. Significantly lower amounts of force were required to displace the tibia for the patellar and quadriceps tendon grafts at 3.76- and 5.34-mm graft diameters. Based on this point, we conclude that grafts below the 7 mm diameter have a higher chance of failure regardless of graft selection.

## Introduction

The ACL originates at the medial side of the lateral femoral condyle and runs an oblique course through the intercondylar fossa. It is the primary restraint to anterior tibial translation and secondary restraint to internal rotation of the weight-bearing and non-weight-bearing knee (1). An anterior cruciate ligament (ACL) injury can result in significant impairment, especially in highly active individuals. Numerous graft sources have been described during the evolution of ACL reconstruction surgery (2, 3). Traditionally, the surgical approach to ACL reconstruction favored the use of allografts or autografts from the patellar tendon, although the hamstrings, and to a lesser degree, the quadriceps tendon have become popular grafts amongst many surgeons and institutions throughout the years (4). Many studies have been conducted to investigate the biomechanical properties of these grafts to guide surgeons in choosing the graft most suitable for their patients (5, 6). More specifically, these studies define biomechanical properties such as tensile strength, elasticity, stiffness, stress and strain responses, and the anatomical measures of these individual grafts. These measures were simulated by the model employed in the study to adequately predict, based on graft selection, how the reconstructed joint functions in consideration of the geometric dimensions employed when designing these individual grafts.

One main limitation of the literature regarding this topic is the need for more data for the quadriceps tendon graft compared to the other graft sources. Additionally, a 2022 systematic review noted a need for studies investigating the effects of diameter on graft selection (4). Considering this second point, two studies investigating this question found that smaller diameter patellar and hamstring tendon autografts are associated with weaker tensile strength and a greater risk of failure (7). However, neither of these studies investigated how the diameters of these grafts would affect the knee mechanics in its working environment. More importantly, no studies have been conducted to determine how diameter affects the strength of the quadriceps tendon. This can be attributed to the fact that the quadriceps were the latest grafts to be popularized by Orthopaedic surgeons (4). Therefore, this study aimed to investigate how diameter affects the biomechanical properties of the quadriceps graft while also comparing it to the “gold standard” patellar tendon graft (8, 9). We hypothesize that when changing the diameters of the quadriceps and patellar grafts, these grafts will tolerate a significant decrease in diameter before they become too weak to meet mechanical requirements for ACL reconstruction, with the patellar tendon capable of withstanding a more substantial reduction in diameter when compared to the quadriceps. Furthermore, we introduced a controlled Lachman test as a measure of knee stability similar to the passive accessory movement test of the knee performed to identify the integrity of the anterior cruciate ligament (ACL). Finally, we aimed to determine how changes in the dimensions of the grafts affect their interaction with various bony and ligamentous features of the reconstructed joint, with an emphasis placed on the graft-epicondylar interface.

## Methods

### Patellar and quadriceps autografts mechanical testing

**Figure 1:**
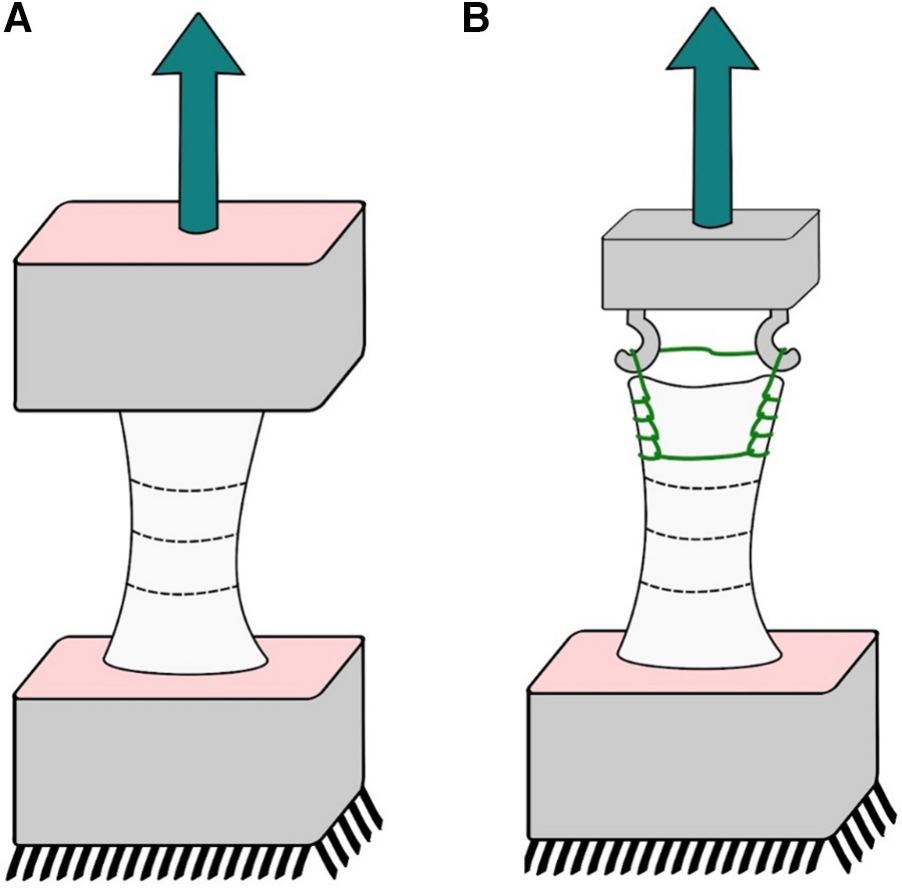
Cadaveric experiments; sketch of our experimental setup: (**A**) patellar tendon and (**B**) quadriceps tendon.

The extensor apparatus was harvested from fresh-frozen, non-irradiated human knees from male and female donors aged 70–90 without any musculoskeletal pathology. Seven quadriceps tendon-patella-patellar ligament-tibial tuberosity complexes were obtained. Each complex was further divided into two complexes (i) a segment of the quadriceps tendon with the proximal half of the patella attached (QT-P); and (ii) the distal half of the patella, the patellar ligament, and a distal insertion of the tibia (P-PL-T) by performing a transverse cut at the midline of the patella using an oscillating saw following a methodology already used by Staubli (10). The exposed tendon and ligament were wrapped with 0.9% NS-soaked gauze throughout the harvesting and placement procedure. To ensure a proper fixation for the experimental testing, the distal ends of each complex were cemented in a metal box with polyester resin. Stabilizing wires were placed at a perpendicular angle through the longitudinal axis of the tendon complex, and the ends of the stabilizing wires were clamped securely to both sides of the box. This procedure was performed for the P-PL-T for both ends (Figure 1A). For the QT-P complex, a Krackow suture was placed within the free tendon end with 3–0 PDS and then secured to the stabilizing wires using multiple suture loops (Figure 1B). The metal boxes were clamped to the materials testing machine (Instron, Instron Corporation, Canton, MA, USA) through a wire loop and a screw. Before testing, 250 N was applied to secure the sutures. Uniaxial tensile testing was performed at an 18 mm/min extension rate.

Using a digital caliper, the exposed tendons and ligaments were marked in the thinner cross-section to measure thickness and width with an initial load of 5 N. The initial length of the tendons and ligaments was an average of 38.5 mm, measured between cemented extremities for the patellar ligament and between the cemented extremity and the suture for the quadriceps tendon. The width was measured in this minor position, and the two adjacent sections were spaced along one-third of the tendon length. The thickness was measured at three equidistant points on these reference positions. The cross-sectional area was evaluated as the width multiplication and the tissue's average thickness. After testing the entire segment, lateral portions were removed to have two remaining sequential samples of two-thirds and one-third in width. On the central one-third, measurements were taken until failure was reached, defined as either separating the tendon complex from the metal housing or separating the housing container from the arm of the machine.

Stress was determined by dividing the force by the corresponding cross-sectional area measurement (see Figure 6). In addition, the Modulus of the tissues was evaluated using linear regression on the obtained measurements.

### Finite element model for the Lachman test

The effective mechanical behavior of the graft and the composition of both material characterization and tensioning are evaluated in a computer-simulated model that best approximates the knee biomechanics. This model was based on the Lachman Test, the most widely used clinical test to determine if the ACL has been torn (11). When building our model, we needed to recreate the structural properties of these grafts by assigning them different inputs for their intrinsic and extrinsic properties according to preselected Young's Modulus values and diameters.

Given the limitations of using cadavers, many labs report various values for the biomechanical properties of the individual grafts being tested (12). To balance these variations in the literature, we chose to use a range of Young's Modulus values for both the patellar tendon and quadriceps tendon grafts to average the results generated from our model to gain a more accurate picture of the investigated effects. We chose to include Young's Modulus values from the literature for the patella tendon of 340.0 MPa (13), 507.4 MPa (14), 565.9 MPa (15), and 597.4 MPa (16), and a value of 263.4 MPa (15) for the quadriceps tendon.

In choosing diameters to test in our experiment, we followed guidelines in the literature, stating that appropriate graft sizes for ACL reconstruction need to restore up to 85%–115% of the native ACL total cross-sectional area (17). We chose our largest diameter of 7 mm based on an experiment that looked at the effect of diameter on the tensile strength of the patellar tendon at diameters of 7, 10, and 15 mm (18). In line with this past study, we further investigated by starting at the lowest data point used in prior studies and expanding to even smaller diameters. Additionally, this value matches the average diameter of the native ACL mid-substance of 7 mm (1). The next smallest diameter of 5.34 mm was chosen based on a value of 5.4 mm diameter reported for the mid substance of the ACL, based on an analysis of female and older age cadavers (average age of 74.8 years) (19). This would allow us to analyze a potential smaller graft for patients with smaller knee geometry. Finally, we chose our smallest diameter of 3.76 mm, which would still align with the required reconstructed area of the native ACL, based on a reconstruction of an ACL with a diameter of 5.4 mm (20).

**Figure 2:**
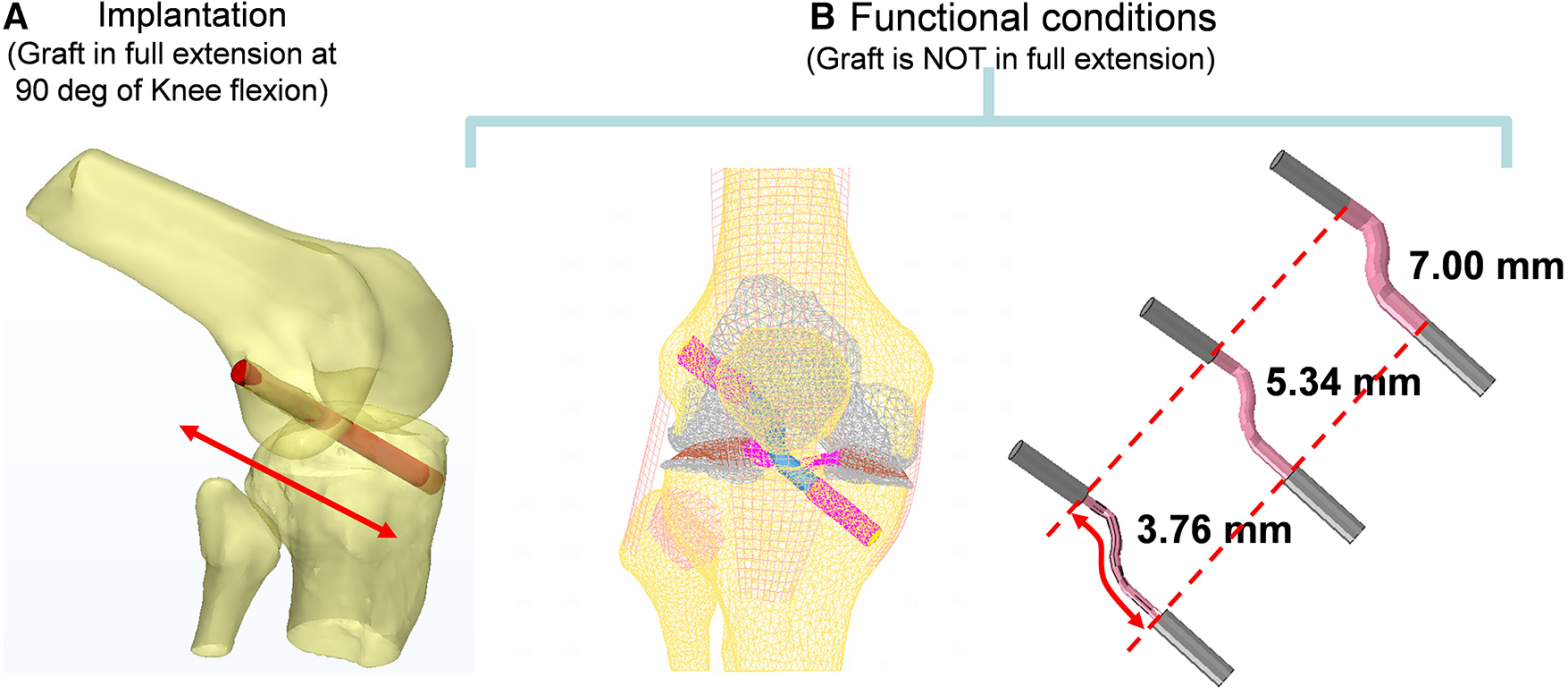
Computer reconstruction of the ACL tunnel during implantation at 90 degrees of flexion using the boolean operation of an 8 mm cylinder in diameter (**A**) and functional conditions (**B**).

The knee bone Geometry was reconstructed from CT scan data using a BrightSpeed (GE Medical Systems) scanner with a slice thickness of 0.625 mm and pixel size of 0.426 mm. The CT images were then imported to Mimics (Materialise, Leuven, Belgium). The tunnels (shown in Figure 2) for the ACL graft were virtually created at 90° of Flexion using a Boolean subtraction with a cylinder 8 mm in diameter (21). Its femoral and tibial positions were based on the studies proposed by Bedi et al. and Gadikota et al. (20, 22). An expert surgeon evaluated the axial slices.

**Figure 3:**
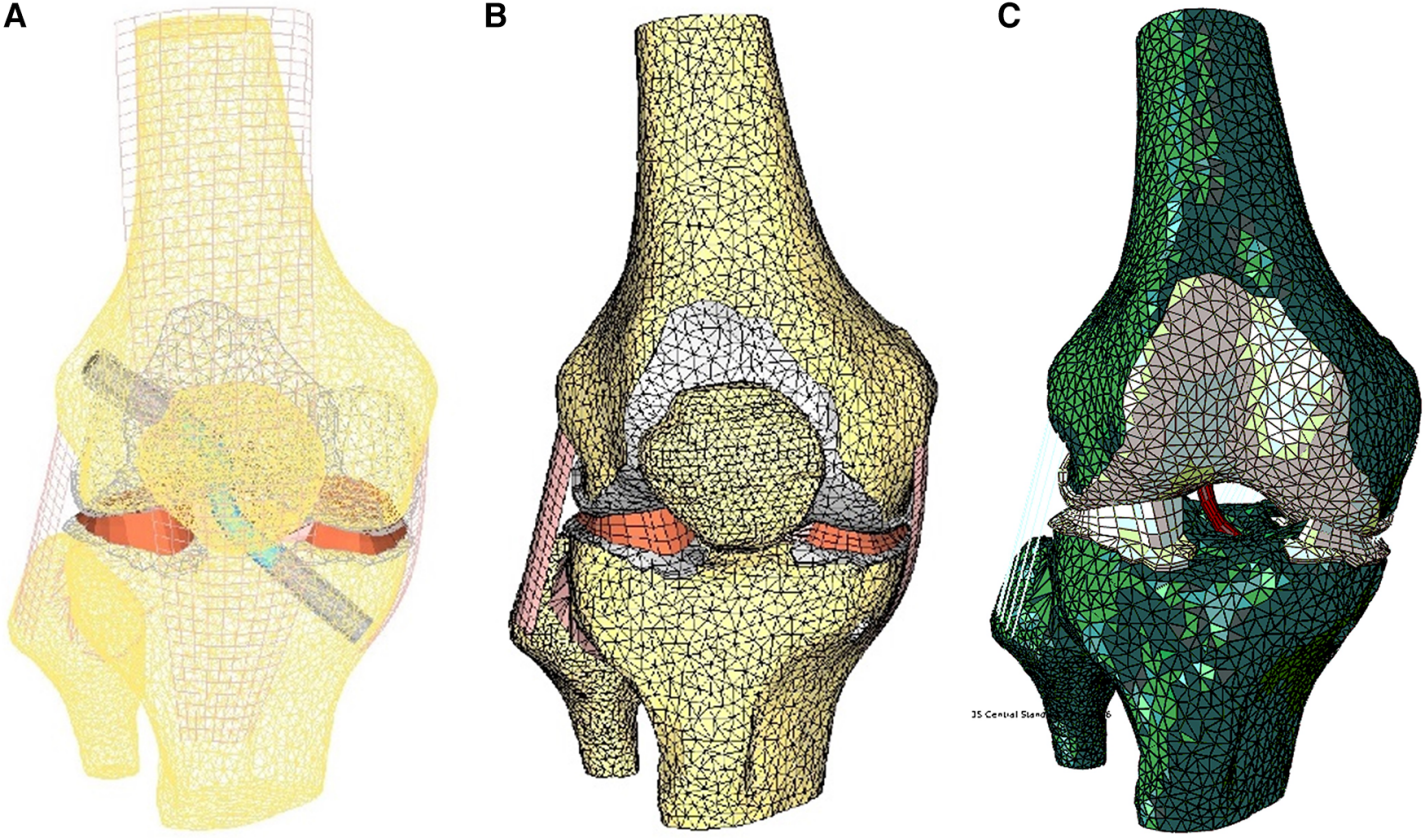
Finite element model of the knee in the unloaded configuration: (**A**) ACL graft simulated, (**B**) 3D external rotation view of cartilage and meniscus layers and collateral ligaments, and (**C**) initial displacement imposed.

Main ligaments were reconstructed following anatomical landmarks on insertion footprints from the literature (23–28). The meniscus was modeled with variable thickness to match the femoral surface in the relaxed position of the CT scan. Considering the aim of this study, the ACL graft was modeled in the knee-flexed configuration as a cylinder and characterized transversely as isotropic (29). The graft in the model is kept in place by two Softsilk 1.5 fixation titanium screws (Smith & Nephew, Andover, MA) 8 mm in Diameter (Figure 3A). All other ligaments are modeled as membrane elements reinforced by truss elements (30–32). The meniscus was modeled as transverse isotropic with an orientation identical to the tibia axis (33). The cartilages (Figure 3B) are created assuming a constant thickness of 2.23 mm for the patella and 2.14 mm for the femur with modulus *E* = 10 MPa and *ν *= 0.45 (34). The cortical bone was modeled with Young's modulus of 17 GPa (35), while the material properties of the trabecular bone were assigned from the CT data (36–38).

**Figure 4:**
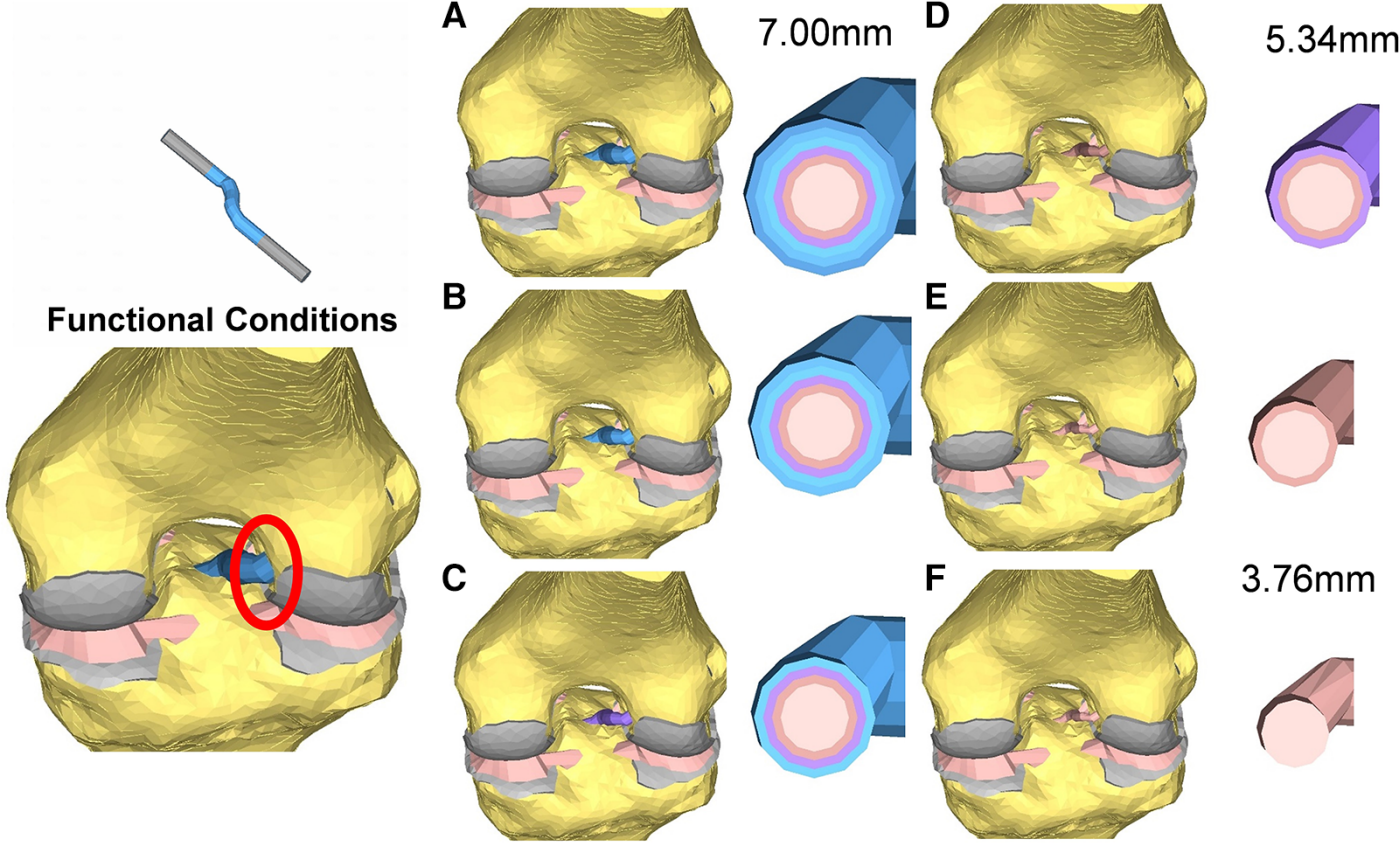
Cross-section view of the reconstructed ACL grafts: the diameters range from 7 mm (**A**) to 3.76 mm (**F**).

The model was simulated, imposing an A-P translation of 15.9 mm, equivalent to the average value reported at the anterior tibial tubercle by Christel et al. (39) for Lachman tests performed on cadaveric specimens (Figure 3C). In addition, six variations in ACL cross-sections from the initial 38.5 mm^2^ were implemented, considering cross-section areas of 22.4 mm^2^ and 11.10 mm^2^ (Figure 4). The boundary conditions were applied to constrain the proximal end of the femur in all degrees of freedom. Additionally, a posterior-anterior displacement was applied to the distal end of the tibia along the projection of the anatomical axis of the femur on the plane sectioning the tibia.

## Results

### Experimental tensile properties

During mechanical testing, no specimen slippage from the fixation device occurred, and no slippage from the fixation device was observed. Patellar ligaments were generally smaller than quadriceps tendons (126.27 and 135.67 mm^2,^ respectively). The cross-sectional area of the patellar tendon was reduced by 10.64% of its initial cross-sectional area at *L*_1_ (first-third of the length) and approximately 34.61% at *L*_2_ (second third of the length). For the quadriceps tendon, the reduction in cross-sectional area for *L*_1_ was 9.77%.

**Figure 5:**
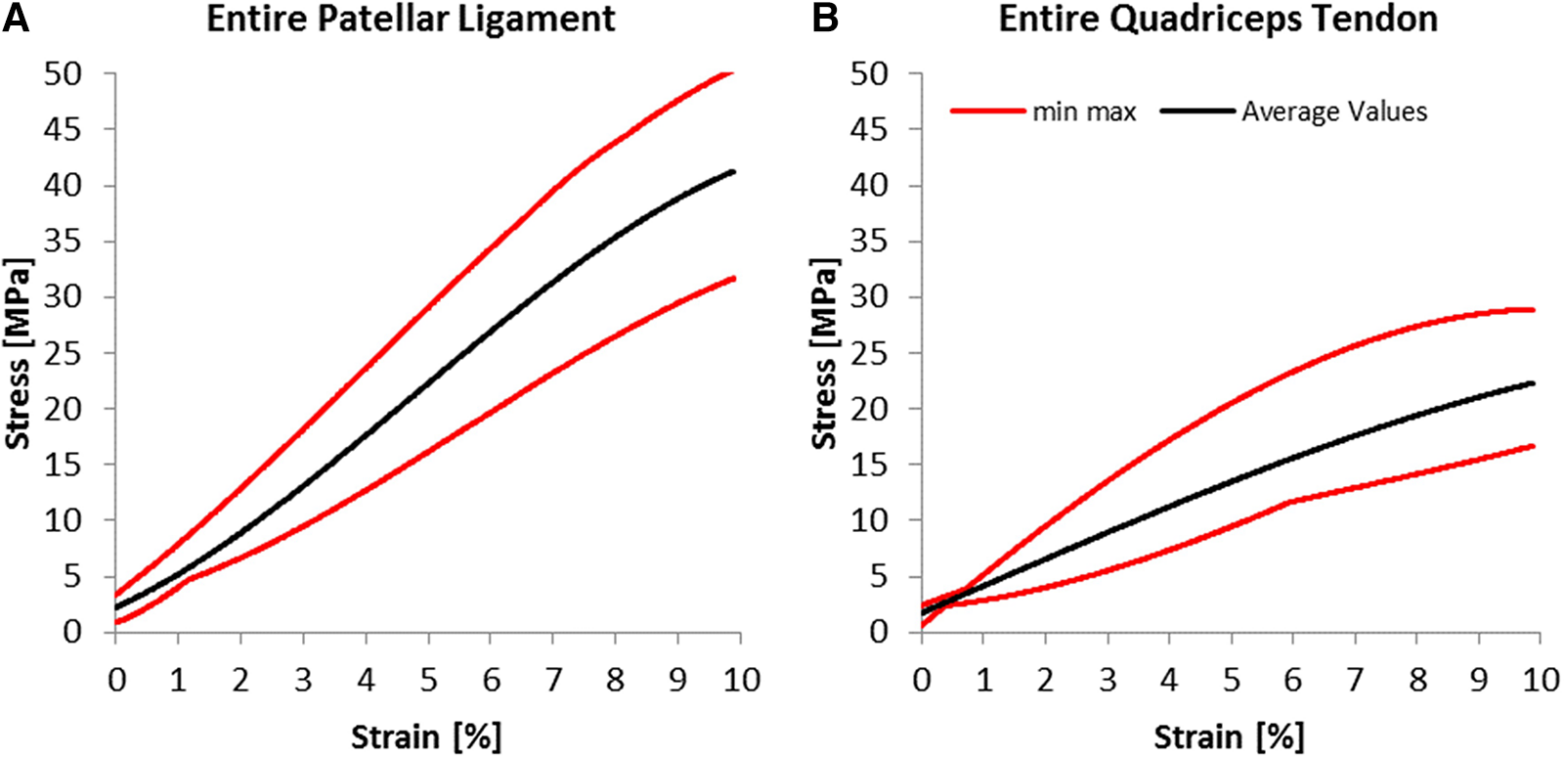
Experimental results; average stress-strain curves and ranges for (**A**) patellar ligaments and (**B**) quadriceps tendon.

**Figure 6:**
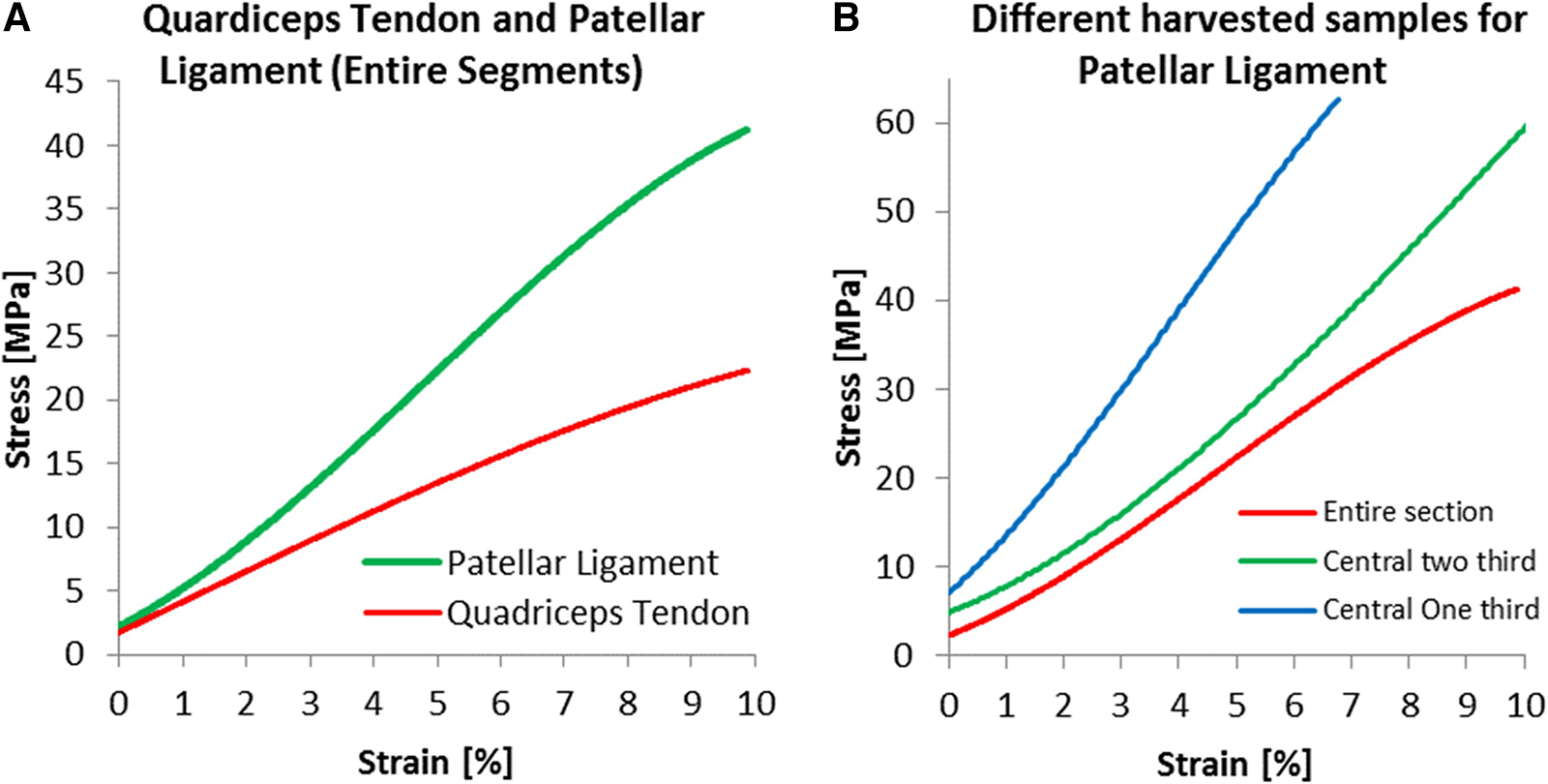
Specific results for harvested dimensions; experimental results for (**A**) entire segments and for (**B**) three harvested dimensions of the patellar ligament.

For the young Modulus evaluated for the entire segment, the quadriceps tendon has shown a standard deviation of 76 MPa, smaller than the 115 MPa estimated for the patellar tendon. Still, as shown in Figure 5, the range between maximal and minimal values recorded for every strain value was more significant for the quadriceps tendon than for the patellar ligament. We have found an increasing modulus with decreasing cross-section (see Table 1). Specifically, the central one-third graft was a drastic 118% stiffer for the patellar ligament than the entire segment. The quadriceps tendon further demonstrates this trend (Figure 6B, Table 1).

**Table 1:** Young moduli from experiments conducted in the current study.

Segment	Patellar ligament			Quadriceps tendon		
Portion	Entire segment	Central two third	Central One third	Entire segment	Central Two third	Central portion
Cross Section [mm^2^]	126.3	83.1	48.5	135.7	105.8	64.3
Modulus [MPa]	409.5	564.6	893.4	176.7	330.5	461.8

The Patellar ligament was stiffer than the Quadriceps tendon (Figure 6A). When comparing the central one-third portion of the tissues, the proportion commonly used for grafts, the patellar ligament was substantially stiffer, with a Modulus of 893.4 MPa, compared to 461.8 MPa for the quadriceps tendon.

### Simulated Lachman test

**Figure 7:**
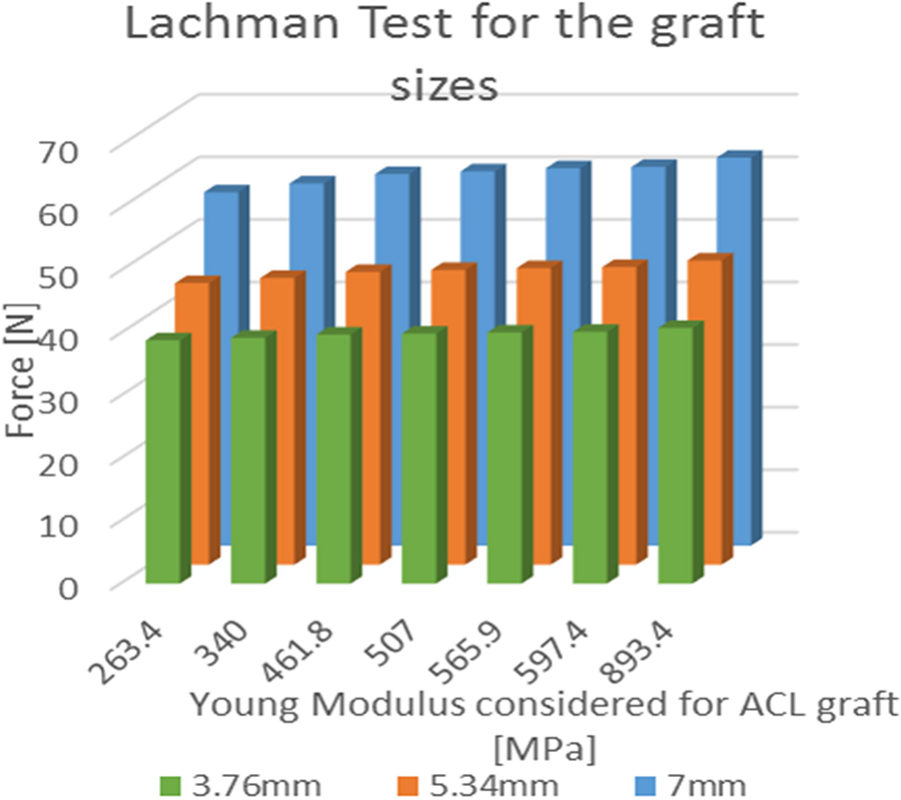
Results of the simulated Lachman test; values of force needed for the A-P displacement according to the young modulus for each of the three considered graft dimensions.

The average force for the Lachman maneuver was 59.48 N ± 1.82 N for the patellar tendon graft with a width of 7.00 mm. For graft sizes of 5.34 mm and 3.76 mm, the force was reduced by 21% and 33%, respectively (Figure 7). When used as a graft, the 7 mm patellar tendon graft was 10.7% stronger than the native ACL. The force calculated using the central portion of the 7 mm quadriceps tendon as a graft was 5.1% greater than the one produced by the native ACL.

In the cases where the quadricep graft sizes were reduced to 5.34 mm and 3.76 mm, the forces were reduced by 27% and 13%. The ANOVA single factor revealed a significant difference between the three graft sizes (*p* < 0.05). In the graft size of 3.76 mm, the patellar tendon graft (893.4 MPa) was 234% stiffer than the quadriceps graft (263.4 MPa), and the force increment was limited to 5.1% (*R*^2^ = 0.946). For graft sizes of 5.34 mm and 7.00 mm, the force increment was evaluated at 8.1% (*R*^2^ = 0.928) and 9.8% (*R*^2^ = 0.909), respectively.

**Figure 8:**
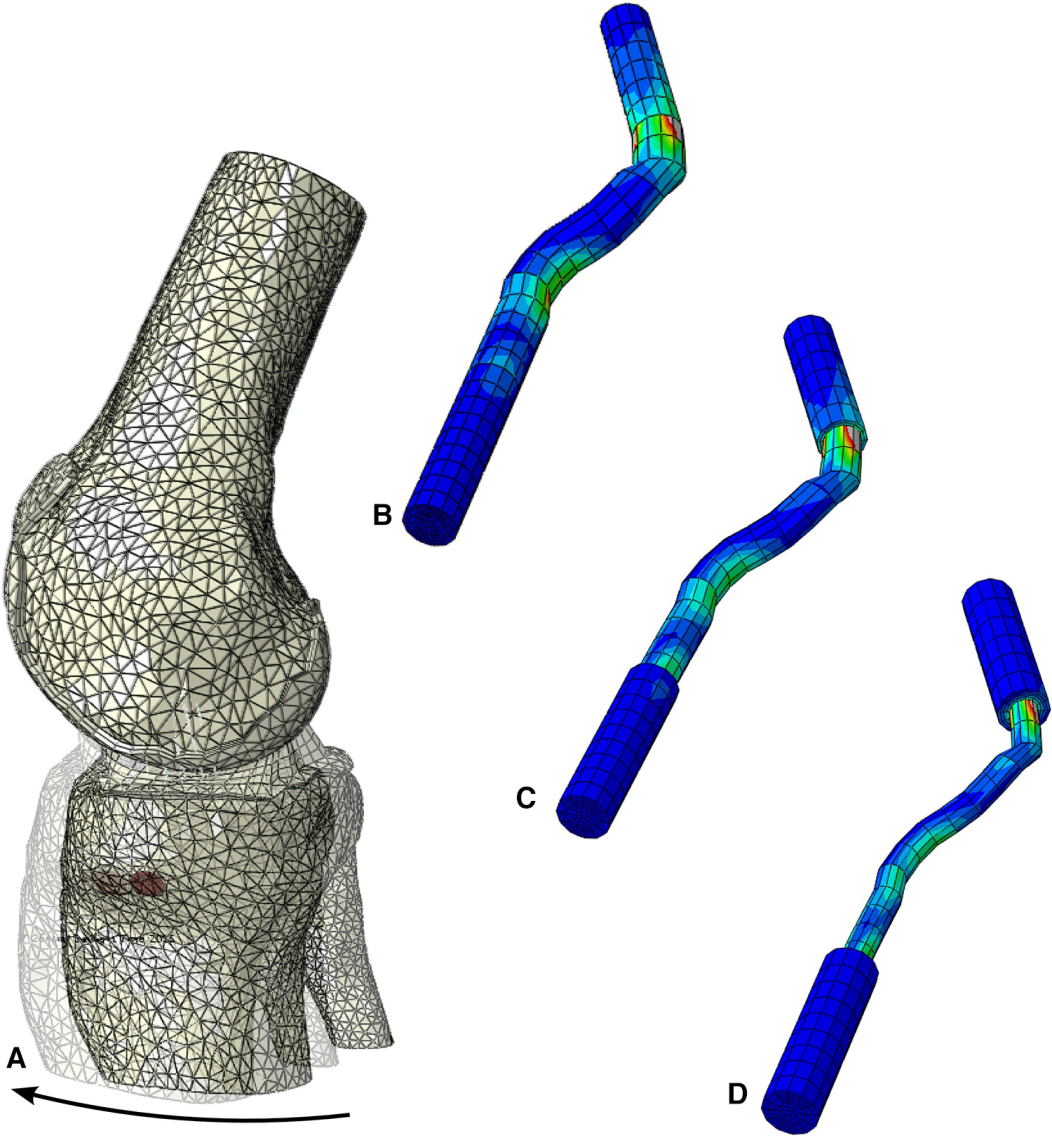
Stress distribution during the simulated Lachman test for the configuration with young modulus of 461 MPa, (**A**) resultant displacement with graft dimension of 7 mm, von mises stress distribution, including the screws, in (**B**) 7 mm, (**C**) 5.34 mm and (**D**) 3.76 mm graft sizes.

We also performed additional tests to investigate the quadriceps tendon (461.8 MPa) as an alternative to the patellar tendon. The maximum stress for the quadriceps tendon with a graft size of 3.76 mm was 43.8 MPa (Figure 8). Increasing the graft dimensions, the maximal stress was significantly reduced to 28.4 MPa and 27.5 MPa for the 5.34 mm and 7.00 mm graft sizes (Figure 8). Following the intercondylar eminence, the stress 10.88 MPa was found for the 7.0 mm graft, whereas the 3.76 mm graft resulted in 8.5 MPa maximal stress. A much more pronounced reduction in stress is seen when comparing the stress risers at the femoral condyle bonding part, where we measured 21.1 MPa and 8.8 MPa for the 7.00 mm and the 3.76 mm grafts. Similar trends in maximum stress were observed for the native ACL and the patellar tendon grafts. However, when comparing values for the maximum stress of the quadriceps tendon to those experienced by the patellar tendon at similar diameters, the patellar tendon grafts in our model showed an average of two times greater maximum stress across relative points of interest on the grafts.

## Discussion

We found that within our model, the patellar tendon outperformed the quadriceps at all graft diameters. When averaging the values for graft diameter and Young's Modulus of the respective graft sources, the patellar tendon required a 10.7% greater force relative to the native ACL to induce tibial displacement compared to 5.1% for the quadriceps. This suggests that the patellar tendon yields more robust grafts for ACL reconstruction at smaller graft diameters. Additionally, the maximum stress for the patellar tendon is at least double that of the quadriceps tendon and about 3–4 times that of the native ACL. This is visualized in Figure 8 by using our modeling results and extracting the maximum stress from the stress response heat map on the tendon. This shows that the patellar tendon better distributes force across the graft and can tolerate more significant stress before deformation.

On the other hand, the patellar tendon has its inherent flaws. The patellar tendon was 234% stiffer than the quadriceps tendon. Additionally, past research found that the Young Modulus evaluated for the patellar tendon is approximately two times greater than that of the quadriceps tendon (see Table 2) (15). This is problematic given an association of excessive stiffness and high Young's Modulus with over-constraining of the knee. A study by O'Brien et al. found that the knee experiences more significant constriction with increasing Young's Modulus. In contrast, Suggs et al. found a correlation between graft stiffness and knee constraining at various flexion angles (16, 40). Additionally, it has been shown that an over-constraining effect is a common postoperative outcome of ACL reconstruction. A study by Robertson et al. that investigated postoperative knee stiffness in 100 patients undergoing primary ACL reconstruction with hamstring autografts found an incidence of postoperative knee stiffness of 12% at six months follow-up (40, 41). Even more, one study found that athletes who have undergone ACL reconstruction with quadriceps tendons experience less stiffness when compared to those who received patellar tendon autografts and performed better in specific compound movements such as squatting (42).

**Table 2:** Young moduli reported in the literature compared to the current study.

Author	Stäubli et al. (10)	Flahiff et al. (13)	Current study	Hashemi et al. (14)	Stäubli et al. (10)	O’Brien et al. (16)	Current study
Segment	Quadriceps tendon	Patellar ligament	Quadriceps tendon	Patellar ligament	Patellar ligament	Patellar ligament	Patellar ligament
Portion	Central portion (CSS 64.6 mm^2^)	Central portion (Width 13.2 mm)	Central portion (CSS 64.3 mm^2^)	Central portion (CSS 20.7 mm^2^)	Central portion (CSS 34.5 mm^2^)	Entire Patellar Ligament (vivo) (CSS 114.8 mm^2^)	Central portion (CSS 48.5 mm^2^)
Ultimate tensile stress [MPa]	38.0	78.4 “800 to 3,000”N	62.2	58.7	69.6	–	68.17
Modulus [MPa]	263.4 (at 200 N) 462.8 (at 800 N)	340.0	461.8	507.4	565.9 (at 200 N) 811.7 (at 800 N)	597.4	893.4

Westermann et al. conducted a similar study using a simulated Lachman model to test graft diameters between 5 and 9 mm (43). However, they designed a graft that restored the joint to ideal proportions in all areas of the knee joint, and that reconstructed the ACL with a graft demonstrating over 150% the strength of the native ACL. In addition, their study showed that anatomically ideal grafts of all sizes could tolerate over 75 N of force before induced tibial displacement. Based on this, we could compare the strength of the grafts within our model relative to the “gold standard” for ACL reconstruction. We found that the 7 mm diameter grafts were the only grafts in our study that showed strength comparable to these ideal grafts, with forces of over 55 N and upwards of -60 N required to induce tibial displacement. Additionally, the grafts below 7 mm failed to reach a strength of even 50 N before tibial displacement. Even more, grafts of these diameters underperformed compared to the native ACL within our model. Given our goal of restoring the native strength of the ACL, grafts below 7 mm diameters do not approximate the strength of an ideal ACL graft and should not be considered for ACL reconstruction.

Regarding the graft size of 3.76 mm, we were not surprised by the results collected. We anticipated this graft dimension would not be suitable for ACL reconstruction but chose to include it in our model to validate the diameter at which the grafts fail. Although this graft size is unsuitable for ACL reconstruction, we aimed to understand how small the graft's diameter can be before it is incompatible with ACL reconstruction, making this data point extremely valuable.

Furthermore, using smaller graft diameters within the experimental design allows the model to better estimate how the total area of the grafts affect their biomechanical properties, while maintaining core constants such as width and length. This allowed us to model smaller graft sizes in the context of understating if smaller grafts are suitable for patients with smaller body frames, while only changing a single dimensional variable, namely diameter, which was already the variable under investigation within our study. A more favorable approach to investigating total area would be to adjust the width and diameters of the grafts while keep these variables within ranges that are commonly employed in surgical practice, however this was out of the scope of the research question. We were able to still gauge the importance of area on the strength of smaller grafts by using graft diameters smaller than those commonly used within clinical practice while also using these graft diameters to formally investigate the extent of which diameter affects ACL graft selection.

Within our study, a few limitations can be identified. Foremost, the cadavers utilized were of a significant age, between 70 and 90. This may have impacted the mechanical properties of experimental characterization. Additionally, the Young's Modulus values found in our study were more significant than those reported in the literature compared to younger cadavers. This finding runs contrary to what has been established in the literature, as it has been well described that the quadriceps undergoes degeneration as the individual ages and should have yielded a weaker response to stress as compared to the quadriceps of younger cadavers tested by other laboratories (44). The different testing conditions employed, namely concerning the load ranges adopted could have impacted the preparation of the grafts, thus explaining why our tissues behaved differentially than those of other researchers. We compensated for this variance by creating a range of Young's Modulus values that would provide us with an average of values to estimate better the actual effect of Young's Modulus on the grafts. Values from the literature were chosen based on studies that followed similar methods to ours. Although we chose to include four Young's Modulus values for the patellar tendon, it is essential to note that only one literature value was used for the quadriceps, given that fewer data exists for this graft source compared to the patellar tendon in the literature.

Next, we used a finite element model to conduct our experiment. We chose to use a model for several key reasons. First, using a model rules out any errors regarding preparing the grafts at their proper diameters. Additionally, variables such as diameter can be easily manipulated within a model, and various results, such as maximum stress and strain across different elements of the reconstructed knee, can be obtained from a single test. This is not attainable when using cadavers. The limitations faced with cadavers are both a lack of accessibility to adequate quantities of cadavers and many inherent flaws that can affect the data collected. These include variables such as how the cadavers were stored, how old the cadavers are, and how the test is designed and carried out. Finally, using a model of the knee in its working environment allowed us to test how diameter affects the mechanics of the joint, something that has yet to be done in prior studies investigating the effects of diameter on ACL grafts.

Currently, no established experimental methodologies exist to validate such interactions between grafts and condyles. Consequently, the conclusions regarding the experimental testing of the tissues have been appropriately separated from the results obtained from the computer model, which overlooks the ACL-bone interaction (45, 46) entirely. The model used within the study primarily serves to investigate the interaction between graft diameter and joint stability but also functions independently to indicate the interaction between the graft and the condyles. Considering this, the measurements taken from the model focused on quantifying the forces distributed across the reconstructed joint at all the anatomical landmarks of the reconstructed joint, with particular consideration of the epicondylar eminence and femoral condyles. Although the validity of this element is limited, the model does quantify the relationship between graft dimensions and the graft-to-condyle interface. This can be used as a preface to further research to investigate this relationship better.

While finite element modeling has its benefits, it is limited in its application and accuracy when compared to research with cadaveric models. Further testing with cadaveric models is highly recommended to conclude what has been found in this study. It would be beneficial to test the functionality of these grafts within cadavers at various diameters with 1 mm increments, starting at 7 mm and going up to 10 mm, to generate finite data to guide surgeons when considering matching functional graft sizes to their patients.

## Conclusion

When designing ACL grafts of a smaller area for use with patients that require smaller than traditional grafts, we found that the patellar tendon provided a stronger graft for knee reconstruction at all diameters. This makes the patellar tendon favorable for use as a graft source in patients requiring smaller grafts. This is further corroborated by the point that the quadriceps tendon undergoes a significantly greater degree of degeneration over time as compared the patellar tendon. For traditional graft sizes, this would not be a factor causing surgeons to favor the patellar tendon over the quadriceps tendon, however when maximally confining graft dimensions in the context of our research question, this element becomes increasingly important to consider.

Assuming that a greater emphasis is placed on the utilization of smaller grafts in target populations, it will be increasingly important to choose a graft source that is stronger and retains a greater functional area as the graft ages. Even more, we conclude based on our model that grafts below 7 mm diameter are unsuitable for reconstruction of the knee. Finally, the interaction we discovered between the condyles and the graft paves the way for future investigations into how graft dimensions can be tailored to the morphometric characteristics of the knee.

## Data Availability

The original contributions presented in the study are included in the article/Supplementary Material, further inquiries can be directed to the corresponding author.
